# Person-centred care in practice: perspectives from a short course regimen for multi-drug resistant tuberculosis in Karakalpakstan, Uzbekistan

**DOI:** 10.1186/s12879-020-05407-7

**Published:** 2020-09-16

**Authors:** Shona Horter, Beverley Stringer, Nell Gray, Nargiza Parpieva, Khasan Safaev, Zinaida Tigay, Jatinder Singh, Jay Achar

**Affiliations:** 1 Médecins Sans Frontières, Chancery Exchange, 10 Furnival Street, London, EC4A 1AB UK; 2 RSSPMCPh&P, Ministry of Health of the Republic of Uzbekistan, Tashkent, Uzbekistan; 3 Republican Phtiziology Hospital #2, Ministry of Health of Karakalpakstan, Nukus, Uzbekistan; 4 Médecins Sans Frontières, Tashkent, Uzbekistan

**Keywords:** Person-centred care, Adherence, Decision-making, Qualitative, MDR-TB, Short-course regimen

## Abstract

**Introduction:**

Person-centred care, an internationally recognised priority, describes the involvement of people in their care and treatment decisions, and the consideration of their needs and priorities within service delivery. Clarity is required regarding how it may be implemented in practice within different contexts. The standard multi-drug resistant tuberculosis (MDR-TB) treatment regimen is lengthy, toxic and insufficiently effective. 2019 World Health Organisation guidelines include a shorter (9–11-month) regimen and recommend that people with MDR-TB be involved in the choice of treatment option. We examine the perspectives and experiences of people with MDR-TB and health-care workers (HCW) regarding person-centred care in an MDR-TB programme in Karakalpakstan, Uzbekistan, run by Médecins Sans Frontières and the Ministry of Health.

**Methods:**

A qualitative study comprising 48 interviews with 24 people with MDR-TB and 20 HCW was conducted in June–July 2019. Participants were recruited purposively to include a range of treatment-taking experiences and professional positions. Interview data were analysed thematically using coding to identify emerging patterns, concepts, and categories relating to person-centred care, with Nvivo12.

**Results:**

People with MDR-TB were unfamiliar with shared decision-making and felt uncomfortable taking responsibility for their treatment choice. HCW were viewed as having greater knowledge and expertise, and patients trusted HCW to act in their best interests, deferring the choice of appropriate treatment course to them. HCW had concerns about involving people in treatment choices, preferring that doctors made decisions. People with MDR-TB wanted to be involved in discussions about their treatment, and have their preference sought, and were comfortable choosing whether treatment was ambulatory or hospital-based. Participants felt it important that people with MDR-TB had knowledge and understanding about their treatment and disease, to foster their sense of preparedness and ownership for treatment. Involving people in their care was said to motivate sustained treatment-taking, and it appeared important to have evidence of treatment need and effect.

**Conclusions:**

There is a preference for doctors choosing the treatment regimen, linked to shared decision-making unfamiliarity and practitioner-patient knowledge imbalance. Involving people in their care, through discussions, information, and preference-seeking could foster ownership and self-responsibility, supporting sustained engagement with treatment.

## Introduction

The standard treatment for multi-drug resistant tuberculosis (MDR-TB) is lengthy, toxic, and insufficiently effective [1], with success rates of just 56% in 2018 globally [2]. The treatment landscape for drug resistant TB is changing, and new drugs are available for the first time in over 40 years [3, 4]. Clinical trials of 15 regimens are underway, with the aim of better tolerability, shorter treatment durations, and new drug combinations, potentially delivering improved treatment regimens [5] and more acceptable models of care.

Person-centred care reflects a holistic model of health that considers the person as central to the process of care, and is flexible to individuals’ needs, choices, and preferences [6, 7]. Patients are situated in their social and biological entirety, with attention given to their identities, subjectivity, environment, and social situation [8, 9]. We define person-centred care as the involvement of people in their care, which includes shared decision-making [6]. Rather than passively receiving health care, the patient is considered an active participant [10]. There is not a one-size-fits-all approach to person-centred care, rather, approaches should be grounded in the reality of how people navigate the pathway from symptoms to cure [11].

The 2019 World Health Organisation (WHO) guidelines for the treatment and management of TB include a short-course regimen (SCR) of older TB drugs lasting 9–11 months, and a longer regimen of 18–20 months that includes new drugs such as bedaquiline [12]. The guidelines emphasise involving people with MDR-TB in their care, and recommend shared decision-making between practitioners and patients regarding the choice of treatment option, for example whether the SCR or a regimen containing new drugs [12]. Uncertainty around the pros and cons of these options requires navigation by patients involved in treatment decisions. Such choices can be complex, and evidence regarding the efficacy, tolerability and implications of various treatment options remains limited. It is important to consider the views of people with MDR-TB regarding their involvement in such choices and of the practitioners implementing the new guidelines. And, since adopting a person-centred approach within the TB response is an internationally recognised priority [7], it is imperative to explore and understand the context-specific preferences and needs of people with MDR-TB regarding their treatment and care.

## Methods

A qualitative study was conducted in Karakalpakstan, Uzbekistan, involving interviews with people with MDR-TB and health-care workers (HCW) during May–July 2019. We aimed to examine perceptions and experiences relating to person-centred care, including shared decision-making for treatment options, to consider how the concept may be realised in practice.

### Study setting

Uzbekistan, situated in Central Asia and with a population of 32.96 million in 2018 [13], had an estimated MDR-TB incidence of 4.7 per thousand population (and TB incidence of 23 per thousand population) in 2018 [2]. Additionally, 18,496 individuals were notified as having TB, and 15% of new TB cases and 34% of previously treated cases had drug resistance [2]. The health system has undergone several major reforms since independence from the Soviet Union in 1991, and is largely structured around decentralised policlinics, as well as hospitals [14]. There is a high coverage of drug sensitivity testing, 88% of notified TB cases were tested with WHO-recommended rapid diagnostics at the time of diagnosis, and MDR-TB treatment coverage was around 50% in 2018 [2]. Reported MDR-TB treatment success is in line with the global average, at 57% [2].

Karakalpakstan is an autonomous region in Uzbekistan, where Médecins Sans Frontières (MSF) and the Ministry of Health have collaboratively provided decentralised, comprehensive TB care since 1998. At the time of this study, project guidelines recommended person-centred care defined as: ambulatory treatment from day one for most drug-sensitive and DR-TB patients; early detection and management of side effects, patient education about disease and treatment (both pre-treatment and as an ongoing process during treatment); adherence counselling provided by counsellors or adherence support nurses; provision of enablers including transport support and food supplements (with social workers where financial instability is identified). Treatment was administered through directly observed treatment (DOT), whereby patients are observed taking their daily medication, and usually travel each day to receive treatment from a treatment location such as a policlinic. Certain patients may receive home-based care, whereby medical personnel travel to the patient’s home to administer treatment, in instances where the individual is unable to travel to the treatment location and based on an assessment of their needs (and consideration of the resources). Treatment for TB and MDR-TB is provided free of charge, funded by the Ministry of Health, Global Fund and MSF.

Historically, approaches to treatment decision-making in the setting have been paternalistic, with doctors deciding on the appropriate course of treatment and care for patients. Shared decision-making for treatment option was to be introduced in 2019 guidelines, implemented shortly after this study. However, it was not clear exactly how treatment decisions would be shared in practice, and new guidelines stated that deciding between the SCR and conventional regimens should be based on patient choice (as well as clinical factors). This therefore offered a pivotal moment to explore perspectives on person-centred care and shared decision-making for treatment option, to ensure that delivery of treatment and care aligns with patients’ priorities and values.

### Participant recruitment

All patient-participants were aged over 18 years and were on or had completed the SCR, or had started on the SCR and transferred out to standard treatment, bar one individual who was on standard treatment (Table 1). Purposive, maximum variation approaches to sampling were adopted to include patients with a wide range of experiences relating to treatment and care, and to balance participants for gender and age (Table 1). Recruitment continued until evidence of data saturation was obtained, whereby adding further participants did not generate new findings. Patients for inclusion were identified from the project SCR database by SH, following stratifying the sample for age, gender and treatment category. Identified patients were then recruited by a gatekeeper from within the project, either a counsellor or a member of the medical team, who introduced the study and asked if the individual would be willing to discuss the possibility of participation.

**Table 1:** Patient characteristics

Characteristic	Number of participants (***n*** = 24)
**Sex**	
Women	12
Men	12
**Age (years)**	
18–24	8
25–34	9
35–44	2
45–58	5
**Treatment category**	
On SCR treatment	15
Completed/cured	5
Treatment failure	3
LTFU	0^a^
Relapse	1
Transferred to or on standard of care	4

SCR = short-course regimen. ^a^It was not possible to recruit people who were lost to follow-up (LTFU); of 6 potential SCR LTFU individuals, 2 were inaccessible (prison, Kazakhstan), 3 were uncontactable, and 1 agreed to meet but did not attend the appointment

HCW participants were recruited purposively to include a range of roles relating to treatment and care delivery: doctors, usually the first point of contact for patients during diagnosis and treatment initiation; nurses, who administer daily medication and provide support throughout treatment; and counsellors who may provide support following diagnosis or during treatment, for example if someone is struggling to sustain engagement with treatment. Again, the number of participants for each employment position were determined based on evidence of data saturation, thereby theoretical approaches to sampling were employed in addition to sampling being purposive. At the time of this study, counselling was focused on supporting those on new drug regimens and was not routinely provided to those engaging with the SCR, and therefore fewer counsellors were recruited for (Table 2). HCW were approached by a research assistant, who introduced the study and invited participation.

**Table 2:** Health-care worker characteristics

Characteristic	Number of participants (***n*** = 20)
**Sex**	
Women	15
Men	5
**Role**	
Doctor	11
Nurse	5
Counsellor	4
**Employer**	
Ministry of Health	12
Médecins Sans Frontières	8

### Data collection and analysis

In-depth interviews were conducted following informed written consent, including for audio recording, to which all participants agreed. Interviews were based on topic guides, which served as reminders for the interviewer (SH), and were flexible and participant-led, with the order of topics depending on the flow of participants’ narratives. Interviews with patients explored their experiences engaging with TB treatment and care, how involvement in care was experienced, and their views about being involved in treatment choices and other ways in which they would like to be involved. Interviews with HCW explored experiences diagnosing people with MDR-TB, how the treatment regimen is decided, HCW views about involving people in choices about their care, and how people are and may be more involved in their care.

Interview topic guides were piloted and adapted. For example, in the first few interviews it was difficult to explore participants’ views about being involved in treatment choices, as the concept was too abstract, therefore scenarios and hypothetical examples were incorporated. Most participants were interviewed once; the option of repeat interviews was discussed at the initial meeting to enable further exploration of emerging topics and explore changes in accounts over time. The generation of data and analytical process were iterative, with analytical thinking and theorising beginning at the point of data collection, which allowed for approaches to be adapted to include more of certain groups of participants, e.g. doctors, and further explore emerging themes.

Data were analysed thematically by SH, using coding to identify emergent themes, patterns, and concepts. Discrepancies from majority themes were actively sought to test the robustness of emerging themes and understand the range of perspectives relating to each category. Transcripts were analysed manually using open, descriptive, in vivo coding, and a coding framework was developed using Nvivo 12 to organise and sort data. Codes and categories were constantly compared within and between participants and findings were raised to a conceptual level, while aiming to remain grounded in participants’ accounts. Attention was paid to the role of the researcher in shaping the data, and analytical codes and categories were shared with NG and BS. Pseudonyms are used to protect participants’ confidentiality, with patient-participants being reflected by P(number), and HCW-participants by HCW(number).

## Results

Interviews were conducted with 24 SCR patient-participants (28 interviews; 3 participants had repeat interviews) and 20 HCW. Patient-participants characteristics are shown in Table 1. HCW were employed by both the Ministry of Health (*n* = 12) and MSF (*n* = 8), with a range of roles (Table 2).

We focus here on findings that emerged relating to aspects of person-centred care, in particular, involvement in choice and care. While we explicitly examined participants’ views and experiences regarding shared decision-making for treatment regimens, findings relating to broader choices and aspects of involvement in care emerged inductively during data analysis. In situating these findings, we draw upon the conceptualisation of person-centred care and present findings around choice and involvement in care separately.

### Perspectives on involvement in choice regarding MDR-TB treatment and care

Most participants appeared unfamiliar with the concept of people with MDR-TB choosing their treatment regimen, either in terms of being involved in the decision-making process, or in stating a preference for their treatment option. This lack of familiarity meant some struggled to imagine the idea of being involved in the choice of treatment, describing the concept as “strange”.

“Here we don’t have it [choice]. I didn’t have it myself. Right, they looked at my papers and they said I was resistant to something. And they told each other that I was going to have SCR. I didn’t understand what the difference was. What was SCR? I didn’t know that there is 2-year treatment with new drugs and 2-year treatment with old drugs.” P19

“Asking these things from the patients is unusual… it will be like a game if they ask from patients… In any case, it will not be like that. No patient will be asked which treatment option he/she prefers.” P02

The decision on treatment regimen was seen primarily as being the responsibility of the doctor, who was perceived as having knowledge and expertise, and therefore being best placed to make the appropriate choice in the interests of the patient. Most patients appeared to trust HCW advice, deferring to their doctor to advise on the appropriate course of action.

“They gave me [treatment] according to my results, then I wasn’t interested, and I didn’t ask. I didn’t take it seriously. I trust the doctors. They will help and do their best for me to get cured… I don’t have any information about it, that’s why I trust the doctors… If it is regarding the treatment, then anyway doctors have more experience than I do… I don’t know what drugs need to be taken, they are educated about it, that’s why I trust them.” P03

“I have never had concerns about it, I trust the doctors. Because I thought that they for sure knew, if they hadn’t known they wouldn’t have done that. Because they studied and they qualified.” P05

Several patients described following the doctors’ orders and the “rules” that were dictated to them.

“I just follow the rules… even if they tell me 9-month or 2-year treatment, I will take what is given.” P18

“We take what doctors prescribe us.” P16

“If the doctors say that this treatment will be correct for you, then there is no choice.” P19

This trust in HCW advice could be reinforced by seeking a second opinion before engaging with treatment, which was described by some participants:

“Here most people consider that whatever doctors say is right… as it is said, “Trust, but check”. Sometimes it happens when I am checked here, I also go there to be checked, in Tashkent. If the disease is too serious. Like TB or oncology… I have been checked not only here, I also went to Tashkent for examination. When I was sure that 9-month fits me then I agreed… in order to be assured that people here made the right decision. Because my life was depending on that.” P04 (university educated)

“Here people trust the doctor and take treatment. Sometimes they go other places without trusting the local ones, thinking that Republican facilities will know better than rayon [rural] level facilities. So, people go to Nukus [capital of Karakalpakstan], not trusting us at the rayon level.” HCW16

However, seeking a second opinion could also lead to individuals engaging with alternative approaches to treating TB. For example, one participant described her husband taking her to Nukus for alternative treatment, saying “the doctors here [rural area] don’t know anything”. She described this as leading to her taking incomplete treatment, which did not cure her disease and which she “regretted”. She said:

“I took hidden treatment… it was not proper treatment… after this treatment I was improved but it was not complete treatment, I just took it for 2-3 months.” P22

Those who did not trust HCW advice were said to be more likely to engage with alternative approaches to managing or curing TB, such as natural healing options:

“They trust in national customs. Some of them. They were telling me that if you drink camel’s milk it can cure many things, and some plants can heal TB. Not trusting the doctors, I think they will go towards them [alternative approaches]. Not wanting to take the drugs of the doctors, they think of being cured naturally.” 02P13

Within a context of unfamiliarity with shared decision-making and established trust-based practitioner-patient relationships, where practitioners hold expertise, several patients had concerns about whether they would be able to decide on their treatment. Many perceived themselves as having insufficient knowledge in comparison to the doctor, fearing that if they were to decide their treatment regimen, they would be responsible for the outcome, and experience blame if they chose the wrong option. This led to discomfort and lack of confidence in making such a choice.

“The doctors never think anything bad for you and they will give whichever one is best, it is better that doctors give treatment. Choosing it myself, I might be mistaken.” P24

“Selecting treatment in TB… when you give a person the right to choose, when he selects what treatment to get, finally, if the patient fails the treatment, then you will say to the patient that the choice was made by him. When the doctor decides on the treatment, then you can bravely say ‘it was the doctor’s decision and it didn’t help’.” 02P04

Choice complexity, and patients perceiving themselves as having insufficient knowledge to choose their treatment regimen could be exacerbated by the shock of receiving a TB diagnosis. Many described the period following diagnosis as difficult, which could undermine their ability to process information and participate in treatment decisions:

“At the start, I was not able to think over because of fear. I just trusted the doctors hoping that they knew.” P05

Some HCW raised concerns that involving patients in the treatment decision would imply that doctors lacked knowledge. HCW felt this could threaten their expertise and damage the trust basis of their relationships with patients.

“Here people are brought up like this, the patient comes, the doctor checks and the doctor gives a prescription… if you tell them that they have two options and to choose one of them, then the patient questions whether the doctor is going to treat me.” HCW14

“Here mostly they [patients] assess us [doctors] because we prescribe, and they will evaluate according to the end result. There might be some notions like ‘If I select myself, why do we need you?’ Or she might think ‘Is she asking me what drugs to prescribe?’. Maybe you are talking about the patients in developed countries.” HCW16

Some HCW felt that patients should not be in involved in treatment decisions as they will just choose the “easiest option” and go for the shorter treatment without comprehending the implications of their choice. This contrasts with the perspectives of patients regarding their preferences for treatment, as the majority stated that while they would prefer a shorter treatment course, it is also important that treatment be effective in achieving cure.

“They will continue choosing a short regimen suitable for them, for them it’s not interesting (they don’t care) whether their lung recovered or not.” HCW01

Several patients asserted that they had a right to know and be involved in the decision-making process, since it relates to their body, health, and treatment; they wanted to be informed about available options and have their preference sought. Some patients who had a high education level asserted wanting to be involved in the treatment choice, though the majority appeared more comfortable with the doctor deciding and preferred to be involved in an informative conversational capacity.

“The person [with TB] will make a selection consciously if he is explained what it is.” 02P04

“Of course, if they tell us like they can treat us with two types of treatment and then you would choose the one which you want for example… if they treat according to our wish it is good for us.” P20

While most patients appeared uncomfortable with the idea of assuming responsibility for treatment decision-making, examples were given of how they enacted choice in other ways regarding their care. For example, most patients described HCW advising them to initiate treatment from hospital, with some even describing this as being “compulsory”; many said that they refused and instead requested initiation of treatment from home or their local clinic.

“I said that I would not be admitted to hospital to start 2-year treatment and I asked the doctor if I could take treatment from home… Then I came home and at the beginning I talked to counsellors and they said that I can take 9-months treatment instead of 2-year treatment. Then I came here [policlinic] and started 9-months treatment.” P13

### Involvement in care: ownership and responsibility

The vast majority of participants highlighted the importance of people with MDR-TB having information and understanding regarding their disease and treatment, for supporting their (sustained) engagement with treatment and care.

“I think the effect of each regimen will be good if the patients have complete understanding and knowledge of the disease and importance of treatment, no matter the regimen.” HCW13

“Ideally patients have a right and they should know about their treatment, what these drugs will do to them, what kind of side-effects they will have or not have, everything about treatment, they have rights.” HCW19

“If they are doing it for me, anyway it is my life, that is why I should know what is happening, what are they doing? It is said that it is hidden, but I want it to be told openly.” P14

Patients described having limited information and understanding, with some wanting more information and giving examples of where they had requested further information from doctors but not received it. This could potentially create an information gap and leave some patients susceptible to seeking information from other sources, for example from other people with MDR-TB.

“I asked what it is. She replied that it was not clear yet, but I had to be hospitalised. She didn’t explain anything in detail… She was treating me like I was a small kid… She didn’t say anything. Maybe she didn’t want to tell me. I didn’t like it, because I want to know when something wrong happens to me. I don’t want the information to be delivered to me via someone.” 02P04

“In [hospital] doctors didn’t give so much information, I myself searched how I can beat TB… maybe they are too busy, there are many patients.” P22

Patients appeared to trust information from other people with MDR-TB above other sources of information. Several patients described information they received from their peers that supported SCR or highlighted the difficulties that people faced with the longer regimen as supporting their engagement with SCR.

“When I was hospital, I found out about it [SCR]. Everyone was saying that if I took those drugs I would be cured.” P08

“I knew about 2 years as my sister-in-law that I mentioned took 2-year treatment. I was glad that I was eligible for 9-month, being worried that I couldn’t take 2 years... I was happy that it was 9-month treatment.” P16

“The information patients pass to each other is more powerful than what doctors or nurses tell them.” HCW02

“There are some educated patients who heard about 9-month SCR and they ask from us the possibility to be enrolled into 9-month SCR. because, in the IPD [in-patient department] they converse with other patients. There are some patients who ask that.” HCW16

It appeared important for patients to have evidence of treatment need and effect, additional to information. This evidence could be experientially conceived, such as through symptoms improving, or achieved through changes in sputum test results being communicated by HCW. Improvements in results appeared to offer evidence of treatment effectiveness, which could be drawn on to motivate sustained treatment-taking. However, results do not always improve, and changes in results or in treatment duration may not always be communicated or retained. Counsellors shared examples of patients being misinformed about their treatment length.

“They don’t know if in this month the results are positive or negative, and the information is not delivered to patients directly from doctors. Even though when patients strictly demand the provision of information on sputum result, it is really difficult to get it… because of doctors’ inattentiveness. When the doctor goes to medical rounds, he goes there unprepared… maybe they are afraid to provide incorrect information and want to have reliable information on hand to patients, not to mix up results.” HCW18

“Most of the time we have difficulties with the relationship between doctor and patient, there is no explanation from doctors’ side, [patients] are angry at the doctor’s behaviour, they [doctors] are shouting like ‘I am responsible for your health, and you should take these drugs which I gave.’ Like… that’s why we are here, counsellors. Many patients, many problems, many misunderstandings… ideally patients should know about their health, their results, ideally. But patients, always they are complaining: ‘the doctor didn’t tell me about my analysis…’ HCW19

Management of information that is shared with patients and misinformation about treatment length were seen as potentially undermining motivation for treatment-taking. Patients were described as preparing and adjusting their mindset to the length of treatment, and could feel betrayed and undermined if a change was not clearly communicated:

“They tell patients that they will take treatment for a short time, they don’t say 2-years… And then the patient sets time for himself and prepares, for example if the patient is going to start 2-year treatment, but the doctors say that it will be 6-months, then anyway after 6-months patients will stop taking the medication, also because they will start feeling OK.” HCW18

Involving people with MDR-TB in their care, through dialogue, information, and seeking their preferences, and ensuring people with MDR-TB have accepted their diagnosis and feel ready for treatment, were seen to increase individuals’ sense of responsibility over their treatment-taking. Several HCW described such responsibility as being important for motivating treatment-taking, facilitating people with MDR-TB prioritising treatment-taking over other areas of life, and being able to continue.

“The treatment is not mandatory here. If you involve and ask patients, then the responsibility will be over the patients… If you don’t put the responsibility on the shoulders of the patients, they start being irresponsible and not taking drugs… If the patient really wants to complete the treatment, the patient will take treatment responsibly and finish treatment.” HCW15

“If the patient has understanding about the disease and drugs, and he knows how to take and what are the side-effects, that patient can take drugs himself.” HCW14

Several participants felt that directly observed treatment (DOT) placed the emphasis of control for drug intake with the observing HCW, rather than the individual taking treatment feeling responsible for their treatment-taking:

“Anyway, doctors were making us take the drugs… They were controlling it. They would be sitting like this and making us drink the tablets.” P05

However, several HCW had concerns that without such control, patients would not be able to adhere and self-motivate their treatment-taking, suggesting that while there is recognition of the value of an individual’s responsibility for their treatment-taking, there are doubts and distrust as to whether this is achievable in reality:

“To have good treatment and to have good outcome. For example, it is needed to stand in front of the patients when they take drugs… There are some patients, adherent ones who tell us that it is very easy not to take drugs if someone doesn’t remind them about taking and not control them strictly.” HCW13

## Discussion

We found that the concept of people with MDR-TB choosing their treatment regimen was unfamiliar, and there was a preference for doctors being responsible for this choice. Patients wanted to be involved in discussions regarding their treatment and care, to have more information and understanding, and have their preferences sought, rather than being responsible for the decision itself. Information, understanding, and involvement in care appeared to increase individuals’ sense of ownership and responsibility for their treatment-taking, which could foster motivation for continuing with it. These findings highlight the importance of considering approaches to person-centred care that are contextually adapted and tailored to individuals’ preferences, comfort, and needs.

Within the rationale of autonomy, choice is assumed to be good as by making choices we become in control of our own lives [15]. However, this binary framing risks overlooking the complex processes involved in individuals’ decision-making [16], which are socially embedded and situated beyond the medical realm, as has been evidenced in the context of HIV [17, 18]. Mol argues that making decisions about our own care can be difficult, with fear and emotion potentially clouding judgement, and with rational, objective choices being near impossible when risks are unknown and the future is uncertain [15]. We found that the concept of shared practitioner-patient treatment decision-making was unfamiliar and abstract in the study context, and that giving patients the responsibility for decisions relating to their treatment may actually cause stress and erode the concept of “good care” [15].

In our study, many patients felt they lacked sufficient knowledge to make an informed choice about their treatment regimen, seeing doctors as best placed to make this choice, and fearing the consequences of making a wrong decision. Evidence from cancer-related research has found that patients can experience anxiety about taking responsibility for the outcome of treatment [19], and want to share the responsibility for decision-making [20]. In our study, patients asserted not feeling comfortable being responsible for treatment decisions, fearing blame if they made the wrong choice. This may be exacerbated by the infectious nature of TB, with treatment failure posing risks for transmission as well as having individual consequences. Additionally, HCW felt that involving patients in decisions would imply that doctors did not know the best course of action, undermining their responsibility and the trust that individuals place in their care.

Many patients described trusting the doctor to decide on their treatment, perceiving the doctor as having their best interests at heart. Practitioner-patient relationships appeared hierarchical, with an asymmetry of knowledge in favour of the doctor, who was seen as the expert and who was deferred to by patients. The power imbued within practitioner-patient relationships is described by Parsons in his conceptualisation of the “sick role”, whereby patients are dependent on medical expertise and authority when facing life-threatening conditions [21]. Where there is an implicit power imbalance due to knowledge asymmetry, patients take a vulnerable position in relation to HCW, and trust may become particularly important in dynamics affecting engagement with treatment and care [22]. Trust is said to be essential for effective therapeutic encounters [23], which becomes heightened in the face of uncertainty, as has been described in the context of cancer treatment decisions [19, 24]. Additionally, evidence regarding vaccine confidence and engagement finds trust to be a crucial aspect of vaccine decision-making [22]. The conceptualisation of person-centred care often prioritises shared decision-making [25], and autonomy is one of the core principles of health care ethics [26]. However, in our study, trust was pervasive in the accounts of people with MDR-TB, appearing to take precedence over having autonomy for treatment decisions.

While participants stated a preference for the doctor being responsible for treatment decisions, patients described the ways in which they enacted choice regarding the location of their treatment initiation, whether ambulatory or hospital-based. Our findings highlight the importance of giving people with MDR-TB the choice about their preferred treatment location, where possible.

Supporting individuals towards self-management, including through taking ownership of health needs [27], and involving people in their care more broadly, are key aspects of person-centred care [6]. In our study, despite health education that is delivered within TB care, knowledge and information about TB and treatment appeared low, with an information gap that people with MDR-TB may fill through seeking information from their peers. The context of low TB knowledge and influence of peer-to-peer information has been described in Uzbekistan [28, 29]. Additionally, we found that patients could be misinformed, for example about the treatment length, which could potentially undermine their motivation for sustained engagement with treatment. Having more information and understanding relating to MDR-TB and treatment appeared to support individuals having a sense of ownership over their health and treatment-taking, fostering self-responsibility and driving individuals to prioritise treatment-taking over other areas of life and to overcome challenges with treatment-taking. Shock at the time of receiving a difficult diagnosis, such as TB, can make it difficult for individuals to receive and process information at this point [30]. Being informed and prepared appeared to support engagement with treatment in our study. However, with TB, the treatment decision must be expedited due to transmission concerns, and therefore information and support may need to continue following treatment initiation.

We found that within DOT approaches the locus of control for drug intake appeared to lie with the observing HCW, rather than with the patient, which may undermine people with MDR-TB having sense of ownership and self-responsibility for their health and treatment-taking. DOT forms the centrepiece for treatment delivery and adherence support in many settings, while lacking a rigorous evidence base and reportedly contradicting patient needs and preferences [6]. HCW had conflicting views about DOT. Most asserted the importance of patients having a sense of responsibility over their treatment, valuing their health and treatment, for enabling their achieving cure, which DOT may contradict through positioning the HCW as responsible for regulation of treatment-taking. However, many HCW had concerns that if they did not “control” patients’ drug intake, there was the risk they would not take treatment as prescribed, and several felt adherence would be worse, even among “adherent patients”, without the presence of DOT. Self-administered treatment for rifampicin-resistant TB in South Africa has been shown to enable integration of treatment-taking into daily life, increasing patients’ autonomy and potentially supporting adherence, among a cohort of clinically stable, adherent patients [31]. As treatment options become shorter and potentially more tolerable and effective, further research should be conducted into approaches to treatment delivery to ensure that they are person-centred.

## Limitations

This research took place before the implementation of TB guidelines that emphasise shared decision-making, which in this context was not common practice and where historically practitioners have made decisions on behalf of patients. Therefore, most people with MDR-TB were unfamiliar with the concept of choice, and found it difficult to imagine or visualise something so abstract. This lack of familiarity influences the study findings and may change as shared decision-making becomes more commonplace. The findings, while likely to be of relevance to similar contexts with a shared recent history, are unlikely to be directly transferrable to substantially different settings. Participants may have perceived the interviewer (SH) as being linked to the health programme, which could have influenced their accounts and made it difficult to access views that were not deemed to be socially desirable. However, SH was independent from the programme, and the findings were reiterated, robust, and fairly critical of the concept of shared decision-making for treatment.

## Conclusions

Our findings highlight that in this context, people with MDR-TB may prefer to be involved in discussions about their treatment and care, and have their preference sought, rather than having responsibility for the treatment choice itself. This finding appeared to be largely linked to unfamiliarity with the concept of shared decision-making, and the knowledge imbalance within practitioner-patient relationships. Patients felt more comfortable deferring to the expertise of HCWs, who were largely trusted to act in their best interests. People with MDR-TB did appear to enact choice regarding the location of treatment, whether ambulatory or hospital-based, and should be involved in this decision.

We found that involving people in their care, through information, discussion, and clear communication regarding treatment expectations and length, could support the engagement of people with MDR-TB, fostering a sense of ownership and self-responsibility for treatment-taking that could drive continuation. This finding may be particularly important in light of the challenges of taking MDR-TB treatment, driving patients to prioritise treatment over conflicting life demands. Programmes should consider ways in which dialogue and communication between practitioners and patients can be strengthened, so individuals can discuss their preferences, questions and concerns regarding their treatment and care. Additionally, patients having evidence of their treatment need and effect may foster motivation for treatment-taking. This should therefore be emphasised within health messaging and communication of changes in sputum results.

## Data Availability

MSF has a managed access system for data sharing that respects MSF’s legal and ethical obligations to its patients to collect, manage and protect their data responsibly. Ethical risks include, but are not limited to, the nature of MSF operations and target populations being such that data collected are often highly sensitive. Data are available on request in accordance with MSF’s data sharing policy (available at: http://fieldresearch.msf.org/msf/handle/10144/306501). Requests for access to data should be made to data.sharing@msf.org.

## Abbreviations

DOT: Directly Observed Therapy
HCW: Health care worker
HIV: Human Immunodeficiency Virus
MDR-TB: Multi-drug resistant tuberculosis
MSF: Médecins Sans Frontières
SCR: Short-course regimen
WHO: World Health Organisation
